# Effect of telecounseling on depressive symptoms in primiparous women during the postpartum period: a systematic review and meta-analysis of randomized controlled trials

**DOI:** 10.3389/fpsyt.2026.1863575

**Published:** 2026-08-28

**Authors:** Mengying Zhou, Hui Hu, Qi Wang, Sheng Zhang

**Affiliations:** Department of Obstetrics, Zhejiang Hospital, Hangzhou, China

**Keywords:** Edinburgh postnatal depression scale, postpartum depression, postpartum period, primiparous women, telecounseling, telehealth

## Abstract

**Background:**

Telecounseling has emerged as a potential strategy to improve access to psychological support during the postpartum period. However, evidence regarding its effectiveness specifically among primiparous women remains limited. This systematic review evaluates the effect of telecounseling interventions on depressive symptoms among primiparous women during the postpartum period.

**Methods:**

PubMed, Scopus, and Web of Science were searched from inception to February 23, 2026. Eligible studies included randomized trials evaluating telecounseling interventions among primiparous postpartum women and reporting depressive symptoms using the Edinburgh Postnatal Depression Scale (EPDS) as continuous outcomes. Random-effects meta-analyses were performed to pool post-intervention mean differences and mean change scores. Statistical heterogeneity was assessed using the I² statistic, and sources of heterogeneity were explored using meta-regression.

**Results:**

Fifteen randomized controlled trials involving 2,297 participants (telecounseling: n = 1,145; control: n = 1,152) were included. The mean age of participants across studies was 28.01 years. Telecounseling was associated with a statistically significant reduction in post-intervention EPDS scores compared with control (z = −6.26, p < 0.001), although substantial heterogeneity was observed (I² = 96.79%). Subgroup analyses demonstrated significant reductions in EPDS scores at 1 week, 1 month, 2 months, 3 months, and 6 weeks, while no significant differences were observed at later time points. Meta-regression indicated that longer follow-up duration was associated with less negative effect estimates, indicating attenuation of the effect favoring telecounseling over time (coefficient = 0.0642 per week, p = 0.003). Thirteen trials were included in the mean change analysis, which also demonstrated a greater reduction in EPDS scores with telecounseling compared with control (z = −4.86, p < 0.001), with substantial heterogeneity (I² = 90.14%). Follow-up duration remained significantly associated with effect size (coefficient = 0.0807 per week, p < 0.001). No significant associations were observed for maternal age, proportion of vaginal delivery, or risk of bias classification. Risk of bias assessment indicated low risk in eight studies and some concerns in seven studies.

**Conclusions:**

Telecounseling was associated with reductions in depressive symptom scores among primiparous postpartum women compared with routine care, although substantial heterogeneity was observed and treatment effects varied across follow-up durations.

**Systematic review registration:**

https://www.crd.york.ac.uk/prospero/, identifier CRD420261333250.

## 1 Introduction

Postpartum depression (PPD) is a common mental health condition that affects women during the postpartum period and may have significant consequences for maternal well-being, infant development, and family functioning (1). The transition to motherhood is associated with substantial psychological and physiological changes, and first-time mothers may be particularly vulnerable to emotional distress due to the demands of adapting to a new parental role and limited prior caregiving experience (2). Untreated postpartum depression has been associated with impaired maternal functioning, reduced mother–infant bonding, and adverse developmental outcomes in children, highlighting the importance of early detection and effective management (3).

Psychological interventions, particularly cognitive behavioral therapy and interpersonal therapy, are considered effective treatments for perinatal depression, and pharmacotherapy may also be indicated depending on symptom severity (4). However, access to conventional mental health services during the postpartum period remains limited due to multiple barriers, including stigma, limited availability of trained providers, time constraints, childcare responsibilities, and logistical challenges (4, 5). These barriers have prompted increasing interest in telehealth-based approaches as alternative methods of delivering mental health care.

Telemedicine and telecounseling interventions, delivered through telephone, internet-based platforms, or mobile applications, have been proposed as accessible strategies for providing psychological support during the postpartum period. Previous systematic reviews have suggested that telehealth interventions may reduce depressive symptoms among postpartum women. For example, Nair et al. (6) reported that most randomized trials of telemedicine interventions demonstrated improvements in maternal depressive symptoms, although methodological limitations such as attrition and lack of blinding were frequently observed. Similarly, Liu et al. (7) and Zhao et al. (5) reported significant reductions in postpartum depressive symptoms following telemedicine-based interventions across randomized controlled trials. A systematic review focusing on postnatal telemedicine interventions also demonstrated modest reductions in depression scores among postpartum women (8). More recently, Pan et al. (9) reported that online cognitive behavioral therapy significantly improved postpartum depressive symptoms, with greater effectiveness observed for interventions of longer duration and those incorporating professional guidance. In a network meta-analysis, Haq et al. (10) further demonstrated that therapist-assisted internet-based cognitive behavioral therapy ranked among the most effective non-pharmacological interventions for postpartum depression.

In addition to intervention effectiveness, telehealth approaches may address disparities in access to mental health services among underserved populations. Technology-based interventions have been proposed as potential tools to improve engagement in populations with historically limited access to traditional care, including ethnic minority groups and individuals in geographically remote settings (11). Broader reviews of postpartum support interventions also suggest that structured counseling and support services, including tele-counseling, may improve maternal psychological outcomes, although the strength of evidence varies across intervention types (12). Furthermore, telehealth-based approaches have been increasingly implemented in perinatal care settings, particularly during the COVID-19 pandemic, with reported benefits in mental health and disease management outcomes (13).

Despite growing evidence supporting telehealth interventions for postpartum mental health, several important gaps remain. First, most existing systematic reviews have evaluated heterogeneous perinatal populations (8, 14–16), including both primiparous and multiparous women, without stratified analyses by parity. Primiparous women may experience distinct psychosocial challenges and adjustment processes compared with multiparous women, which may influence treatment response and clinical outcomes. Second, prior reviews have often included a broad range of telehealth modalities and outcome measures, limiting the ability to evaluate specific effects of telecounseling interventions using standardized depression scales (5, 9, 17, 18). Finally, existing syntheses have not consistently distinguished between post-intervention symptom severity and change in depressive symptoms from baseline, which may provide complementary perspectives on treatment response.

To address these gaps, the present systematic review and meta-analysis aimed to evaluate the effect of telecounseling interventions on depressive symptoms among primiparous women during the postpartum period using the Edinburgh Postnatal Depression Scale (EPDS) as a standardized outcome measure. This study also sought to examine treatment effects using both post-intervention mean differences and mean change analyses and to explore potential sources of heterogeneity through meta-regression. By focusing specifically on primiparous women and standardized outcome measures, this review aims to provide a more targeted assessment of telecounseling effectiveness in this population.

## 2 Methods

### 2.1 Study design and reporting standards

This systematic review and meta-analysis was conducted to evaluate the effect of telecounseling on depressive symptoms among primiparous mothers during the postpartum period. The review methodology was defined *a priori*, and the study protocol was registered on PROSPERO (CRD420261333250). The study was conducted in accordance with the Preferred Reporting Items for Systematic Reviews and Meta-Analyses (PRISMA) guidelines.

### 2.2 Search strategy

A comprehensive literature search was performed on February 23, 2026, using the electronic databases PubMed, Scopus, and Web of Science. The search strategy combined controlled vocabulary and free-text terms related to telecounseling modalities, primiparous or first-time mothers, postpartum period, depression, and randomized controlled trials. The PubMed search strategy included terms related to telecommunication-based interventions (e.g., telecounseling, telehealth, telemedicine, internet-based interventions, mobile applications), population descriptors (primiparous, first-time, or new mothers), postpartum-related terms, depression-related terms including the EPDS, and randomized controlled trial filters. The full search strategy is provided in the **Supplementary Material**. These three databases were selected as complementary, multidisciplinary sources that together provide broad coverage of the biomedical, nursing, and psychological literature relevant to this topic, consistent with Cochrane Handbook guidance recommending that at least two relevant databases be searched (19). PubMed indexes MEDLINE, while Scopus and Web of Science capture a large proportion of records that would otherwise be retrieved from Embase, including conference proceedings and journals not indexed in MEDLINE. Embase was not searched because it was not available through our institutional subscription. To reduce the likelihood of omitting eligible trials, the database search was supplemented by manual screening of the reference lists of all eligible studies and relevant systematic reviews.

To ensure completeness, manual screening of reference lists from eligible studies and relevant systematic reviews was conducted to identify additional studies. No restrictions were applied regarding publication year. Only studies published in English were included.

### 2.3 Eligibility criteria

Studies were included according to the following criteria:

- Population: Primiparous women or first-time mothers during the postpartum period.
- Intervention: Telecounseling defined as structured psychological or counseling-based interventions delivered remotely via telecommunication technologies (e.g., telephone, internet-based platforms, or mobile applications). To be eligible, an intervention had to include an interactive, individualized psychological or supportive counseling component delivered or guided by a trained provider (e.g., structured counseling, cognitive behavioral techniques, behavioral activation, problem-solving, or interactive peer or professional support). Programs that also contained an educational or psychoeducational element were eligible provided that this interactive counseling component was present.
- Comparator: Standard care, usual care, or alternative non-telecounseling interventions.
- Outcome: Depressive symptoms measured using the Edinburgh Postnatal Depression Scale (EPDS) reported as continuous data.
- Study design: Randomized controlled trials.

Studies were excluded if they evaluated educational or informational interventions without a counseling component (i.e., interventions limited to the one-way provision of information or didactic education, such as standalone leaflets, automated informational messages, or breastfeeding or parenting instruction, with no interactive or individualized psychological support), included mixed populations of primiparous and multiparous women without separate subgroup data, reported depression outcomes only as binary measures without continuous EPDS data, did not provide sufficient quantitative data for analysis, were economic or cost-effectiveness evaluations, or were non-randomized designs.

### 2.4 Study selection

All identified records were imported into reference management software and duplicates were removed. Titles and abstracts were screened for eligibility, followed by full-text review of potentially relevant studies. Eligibility decisions were made based on predefined inclusion criteria. When multiple reports of the same study were identified, data were extracted from the most complete report. Discrepancies during study selection were resolved through discussion.

### 2.5 Data extraction

Data were extracted using a standardized data collection form. Extracted information included study characteristics, participant characteristics, intervention details, comparator characteristics, follow-up duration, and EPDS scores reported as means and standard deviations at baseline and post-intervention time points. When multiple follow-up time points were reported, predefined time points were selected for quantitative synthesis to avoid duplication of participants.

### 2.6 Risk of bias assessment

The methodological quality of included randomized controlled trials was assessed using the revised Cochrane Risk of Bias tool for randomized trials. Studies were evaluated across domains including the randomization process, deviations from intended interventions, missing outcome data, outcome measurement, and selective reporting. Each study was categorized as having low risk of bias, some concerns, or high risk of bias according to established criteria.

### 2.7 Statistical analysis

Quantitative synthesis was performed using random-effects meta-analysis to account for potential between-study variability. Continuous outcomes were pooled as mean differences (MDs) with corresponding 95% confidence intervals.

For studies reporting both baseline and post-intervention EPDS scores, change scores were calculated as the difference between post-intervention and baseline values within each study arm. The standard deviation of change scores was estimated using established methods incorporating an assumed pre–post correlation coefficient. A correlation coefficient of 0.5 was used for the primary analysis, and sensitivity analyses were conducted using alternative correlation values to evaluate robustness of the findings. For studies reporting only post-intervention data, effect estimates were calculated using post-intervention mean differences.

Statistical heterogeneity was assessed using the Cochran Q test and quantified using the I² statistic. Sources of heterogeneity were explored using meta-regression analyses examining prespecified covariates, including mean maternal age, proportion of participants with vaginal delivery, duration of follow-up (weeks), country of study (with China as the reference category), and risk of bias classification (low risk vs. some concerns).

Small-study effects and potential publication bias were evaluated using funnel plot inspection and Egger’s regression test. Galbraith plots were generated to identify potential outlier studies and to assess heterogeneity patterns.

All statistical analyses were conducted using Stata version 18 (StataCorp LLC, College Station, TX, USA).

## 3 Results

### 3.1 Study selection

The literature search identified a total of 324 records across the electronic databases, including 105 records from PubMed, 140 from Scopus, and 79 from Web of Science. After removal of 163 duplicate records using reference management software, 161 records remained for title and abstract screening. Of these, 45 records were excluded based on predefined eligibility criteria.

**Figure 1:**
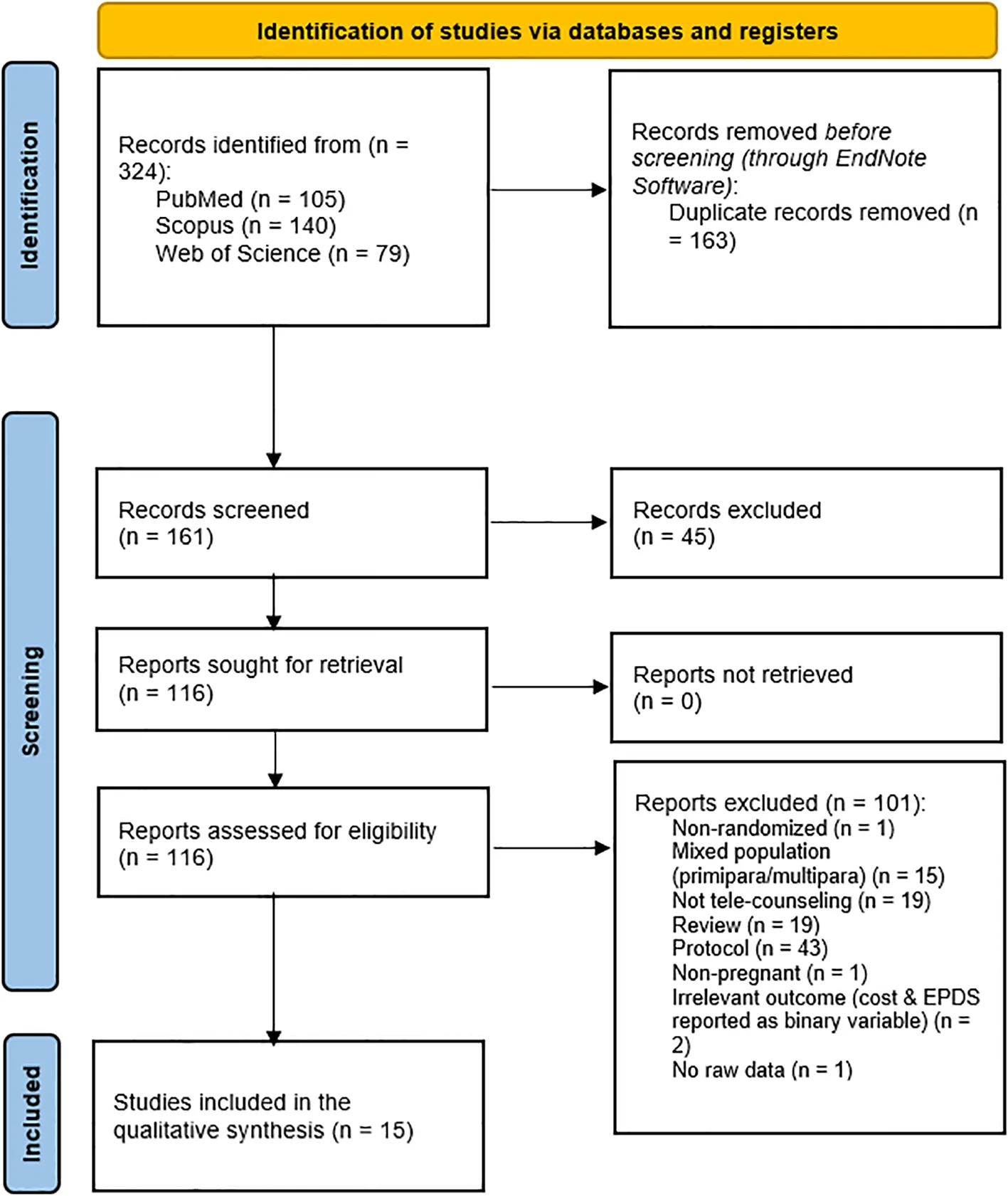
PRISMA Flow diagram.

A total of 116 reports were sought for full-text retrieval, all of which were successfully obtained and assessed for eligibility. Following full-text review, 101 reports were excluded. Reasons for exclusion included non-randomized study design (n = 1), inclusion of mixed populations of primiparous and multiparous women without separate subgroup data (n = 15), interventions not meeting the definition of telecounseling (n = 19), review articles (n = 19), study protocols (n = 43), non-postpartum populations (n = 1), reporting of depression outcomes as binary measures or cost-related outcomes rather than continuous EPDS scores (n = 2), and insufficient quantitative data for analysis (n = 1). Ultimately, 15 studies met the eligibility criteria and were included in the qualitative synthesis (20–34). The study selection process is summarized in **Figure 1**.

### 3.2 Characteristics of included studies

A total of 15 randomized controlled trials involving 2,297 participants were included in the qualitative synthesis, comprising 1,145 participants in the telecounseling groups and 1,152 participants in the control groups. The characteristics of the included studies are summarized in **Table 1**. The mean age of participants across studies was 28.01 years, with reported study-level mean ages ranging from 23.05 to 31.65 years.

**Table 1:** Characteristics of included randomized controlled trials evaluating telecounseling for postpartum depression in primiparous women.

Author (YOP)	Design	Protocol	Blinding	LTFU (%)	Country	YOI	Sample		Age (yr) - mean (SD)		FU
							Tele	Ctrl	Tele	Ctrl	
Suchan (2022) (32)	RCT	NCT04012580	–	16.67	Canada	Sep 2019 - Mar 2020	28	32	31.18 (4.31)	30.53 (4.32)	6 mo
Koç (2024) (27)	RCT	NCT06070168	Single	9.1	Turkey	Oct 2023 - Feb 2024	50	50	25.18 (4.08)	25.58 (4.47)	6 wk
Sawyer (2019) (31)	RCT	ACTRN12616001732471	Single	15.04	Australia	Mar - Jun 2017	54	57	31.1 (5)	32.2 (4)	6 mo
Zhang (2023) (34)	RCT	ChiCTR2000033154	Triple	3.39	China	May 2020 - Mar 2021	118	118	27.28 (3.10)	27.42 (2.93)	3 mo
Eskandari (2025) (23)	RCT	–	Single	6.45	Iran	Oct 2023 - Aug 2024	31	31	23.14 (5.9)	23 (4.97)	2 mo
Chan (2019) (20)	RCT	HKUCTR-2024	Single	17.8	China	2017 - 2018	330	330	31.3 (4.6)	31.2 (4.5)	1 mo
Coo (2024) (21)	RCT	NCT04847076	Single	18.75	Chile	Feb 2021 - Apr 2022	59	57	25.56 (4.1)	24.98 (5.2)	5 mo
Huang (2021) (24)	RCT	ChiCTR2000033154	Single	10	China	May-Oct 2020	20	20	27.15 (3.15)	27.35 (3)	3 mo
Jiao (2019) (25)	RCT	ISRCTN45202278	–	8.8	Singapore	Oct 2016 - Aug 2017	68	68	31.1 (3.8)	30.3 (3.7)	6 mo
Nguyen (2024) (29)	RCT	NCT06053515	–	6.45	USA	Jan - Feb 2023	15	14	31.7 (30–35)	31.6 (26–35)	3 mo
Velioğlu (2024) (33)	RCT	NCT06443801	–	0	Turkey	Mar 2023 - Mar 2024	60	60	27.15 (3.84)	29.87 (3.78)	1 wk
Kamalifard (2013) (26)	RCT	–	Single	4	Iran	2012	50	50	24.14 (3.86)	24.34 (4.34)	8 wk
Dol (2022) (22)	RCT	NCT04730570	Single	12	Canada	Jan - Aug 2021	34	35			6 wk
Öztoprak (2023) (30)	RCT	NCT05225987	Single	4.7	Turkey	May-Dec 2021	31	30	22.52 (3.32)	23.57 (3.41)	3 mo
Ngai (2015) (28)	RCT	–	Single	7.3	China	Jul 2012 - Mar 2014	197	200	31.1 (3.8)	30.4 (4.4)	6 mo

RCT, randomized controlled trial; USA, United States of America; YOP, year of publication; YOI, year of investigation; LTFU, losses-to-follow-up; Tele, telecounseling; Ctrl, control; yr, year; SD, standard deviation; FU, follow-up; mo, month; wk, week.

The included studies were conducted across multiple geographic regions. China represented the most frequently contributing country (n = 4), followed by Turkey (n = 3) and Canada and Iran (n = 2 each), while Australia, Chile, Singapore, and the United States each contributed one study. Follow-up durations varied across studies, ranging from 1 week to 6 months. The most commonly reported follow-up periods were 3 months and 6 months (n = 4 each), followed by 6 weeks (n = 2), with other time points reported in single studies.

Interventions consisted of telecounseling delivered through various remote communication modalities, while control groups generally received routine postpartum care without the telecounseling intervention. Due to the nature of the interventions, blinding of participants and personnel was limited. Ten studies were conducted using single-blind designs, and one study reported a triple-blind design. Study protocols were available for 12 trials.

**Figure 2:**
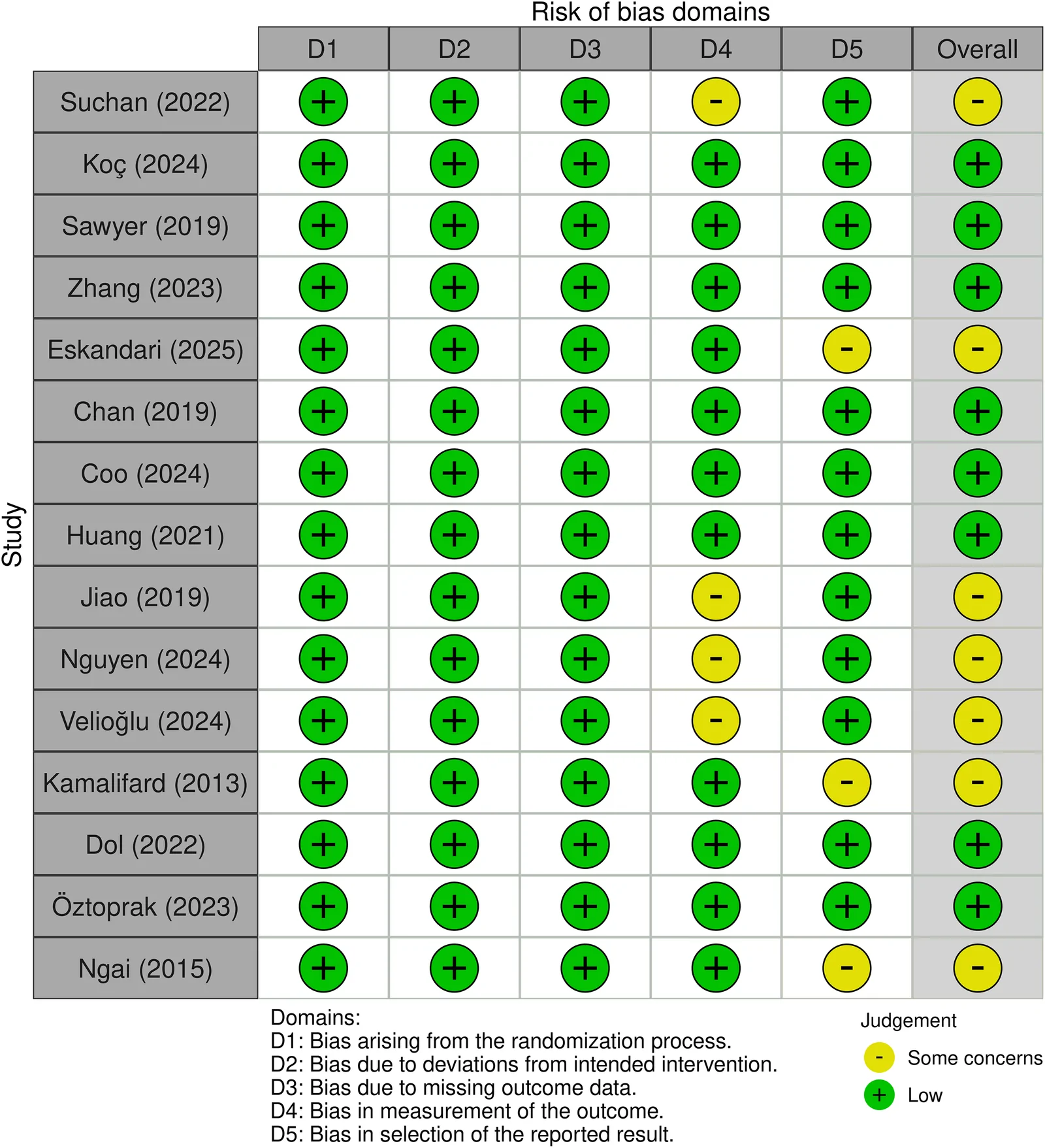
A summary of the risk of bias of included trials.

Loss to follow-up ranged from 0% to 18.75% across studies, with a mean attrition rate of 9.36%. Risk of bias assessment indicated that eight studies were judged to have low risk of bias, while seven studies were classified as having some concerns. No study was rated as having a high risk of bias. Consequently, the risk-of-bias traffic-light plot (**Figure 2**) contains only low-risk (green) and some-concerns (yellow) judgements, and no high-risk (red) ratings appear because none were assigned.

### 3.3 Effect of telecounseling on depressive symptoms (post-intervention mean difference)

A total of 15 randomized controlled trials were included in the quantitative synthesis evaluating post-intervention EPDS scores. Across all follow-up time points, telecounseling was associated with a statistically significant reduction in depressive symptom scores compared with control conditions (z = −6.26, p < 0.001). However, substantial between-study heterogeneity was observed in the overall analysis (I² = 96.79%, τ² = 1.61), indicating considerable variability in effect estimates across studies.

#### 3.3.1 Analysis by follow-up duration

**Figure 3:**
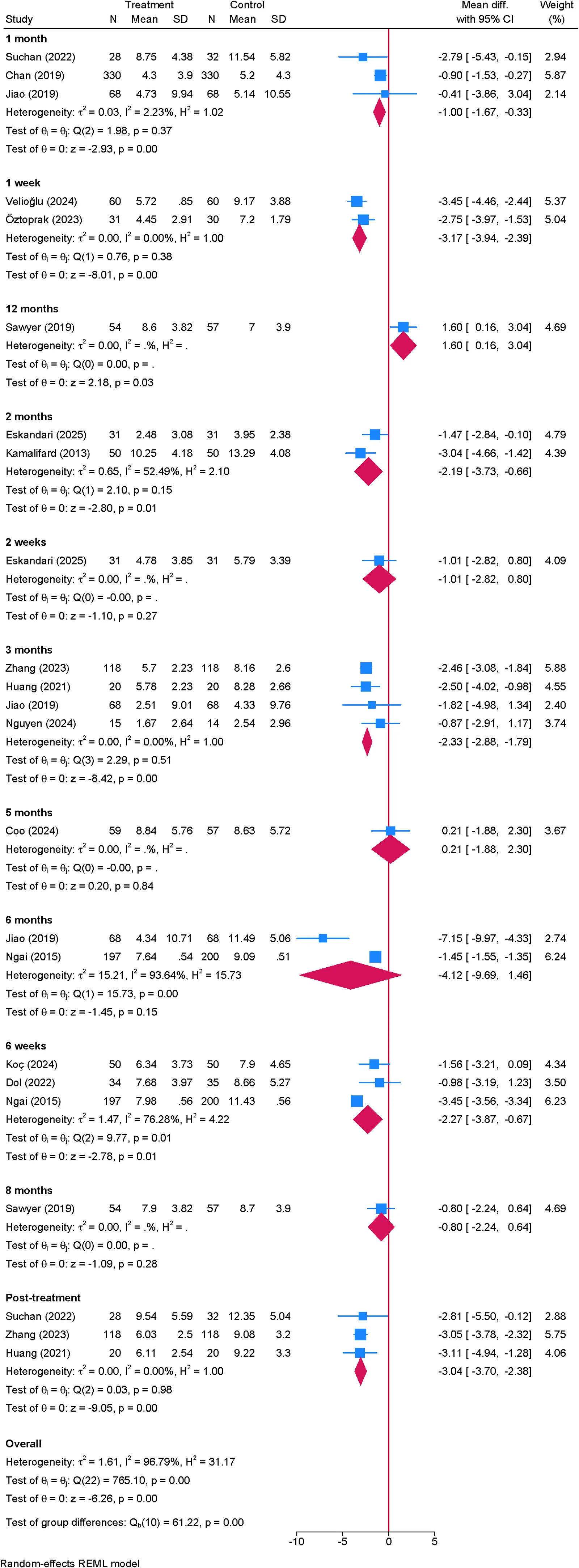
Forest plot of post-intervention mean difference in EPDS scores.

Subgroup analyses according to follow-up duration demonstrated variability in effect estimates across time points (**Figure 3**). At 1 month, telecounseling was associated with a reduction in EPDS scores compared with control (MD = −1.00, 95% CI −1.67 to −0.33; p = 0.003) with minimal heterogeneity (I² = 2.23%). Similarly, at 1 week, telecounseling showed a statistically significant reduction in depressive symptoms (MD = −3.17, 95% CI −3.94 to −2.39; p < 0.001) with no observed heterogeneity (I² = 0%).

At 2 months, the pooled estimate favored telecounseling (MD = −2.19, 95% CI −3.73 to −0.66; p = 0.01), although moderate heterogeneity was present (I² = 52.49%). At 3 months, telecounseling remained associated with lower EPDS scores (MD = −2.33, 95% CI −2.88 to −1.79; p < 0.001) with no observed heterogeneity.

At 6 weeks, the pooled estimate also favored telecounseling (MD = −2.27, 95% CI −3.87 to −0.67; p = 0.01), with substantial heterogeneity across studies (I² = 76.28%). In contrast, no statistically significant differences between groups were observed at 2 weeks (p = 0.27), 5 months (p = 0.84), 6 months (p = 0.15), or 8 months (p = 0.28). At 12 months, the pooled estimate favored the control group (MD = 1.60, 95% CI 0.16 to 3.04; p = 0.03), although this estimate was derived from a single study.

A test for subgroup differences across follow-up durations indicated statistically significant differences between time points (Qb = 61.22, p < 0.001), suggesting that the magnitude of the intervention effect varied according to follow-up duration.

#### 3.3.2 Small-study effects and heterogeneity

Visual inspection of the funnel plot suggested some asymmetry in the distribution of effect estimates (**Supplementary Figure 1**); however, no significant risk of bias was observed (p = 0.8693).

The Galbraith plot demonstrated dispersion of several studies outside the confidence bounds, indicating potential sources of heterogeneity and variability in study-specific effect estimates (**Supplementary Figure 2**).

#### 3.3.3 Meta-regression analysis

No statistically significant association was observed between effect estimates and mean maternal age (coefficient = 0.0493, p = 0.652) or proportion of vaginal delivery (coefficient = −0.0560, p = 0.243). Similarly, risk of bias classification (some concerns vs. low risk) was not significantly associated with effect size (coefficient = −0.9035, p = 0.147) (**Table 2**).

**Table 2:** Meta-regression analysis of the determinants of post-intervention EPDS scores (telecounseling vs. control).

Characteristic	Coefficient	P	2.5% CI	97.5% CI
Age (per year)	0.0493	0.6520	-0.1648	0.2635
Vaginal delivery (per % increase)	-0.0560	0.2430	-0.1500	0.0380
Country (Reference: China)				
Turkey	-0.3576	0.6780	-2.0457	1.3306
Canada	0.2259	0.8320	-1.8663	2.3182
Singapore	-1.1376	0.3440	-3.4919	1.2167
Iran	0.4757	0.5990	-1.2976	2.2490
Australia	2.7244	0.0070	0.7298	4.7191
Follow-up (per week)	0.0642	0.0030	0.0226	0.1059
Quality (Some concerns vs. Low risk)	-0.9035	0.1470	-2.1260	0.3189

P, P-value; CI, confidence interval; EPDS, Edinburgh Postnatal Depression Scale.

Using China as the reference category, no statistically significant differences in effect estimates were observed for studies conducted in Turkey (p = 0.678), Canada (p = 0.832), Singapore (p = 0.344), or Iran (p = 0.599). However, studies conducted in Australia demonstrated significantly more positive (less negative) effect estimates in EPDS scores compared with studies conducted in China (coefficient = 2.7244, 95% CI 0.7298 to 4.7191; p = 0.007).

Duration of follow-up was significantly associated with effect estimates, with increasing follow-up duration associated with more positive (less negative) effect estimates (coefficient = 0.0642 per week, 95% CI 0.0226 to 0.1059; p = 0.003).

Overall, these findings indicate that follow-up duration and study location may contribute to heterogeneity in observed treatment effects, whereas maternal age, mode of delivery, and study quality were not significantly associated with effect estimates.

### 3.4 Effect of telecounseling on depressive symptoms (mean change analysis)

A total of 13 eligible randomized controlled trials were included in the meta-analysis of change in EPDS scores from baseline to follow-up. Using a random-effects REML model, telecounseling was associated with a statistically significant greater reduction in EPDS scores compared with control (z = −4.86, p < 0.001). However, substantial heterogeneity was observed across studies (τ² = 2.16; I² = 90.14%), indicating considerable variability in effect estimates.

#### 3.4.1 Analysis by follow-up duration

**Figure 4:**
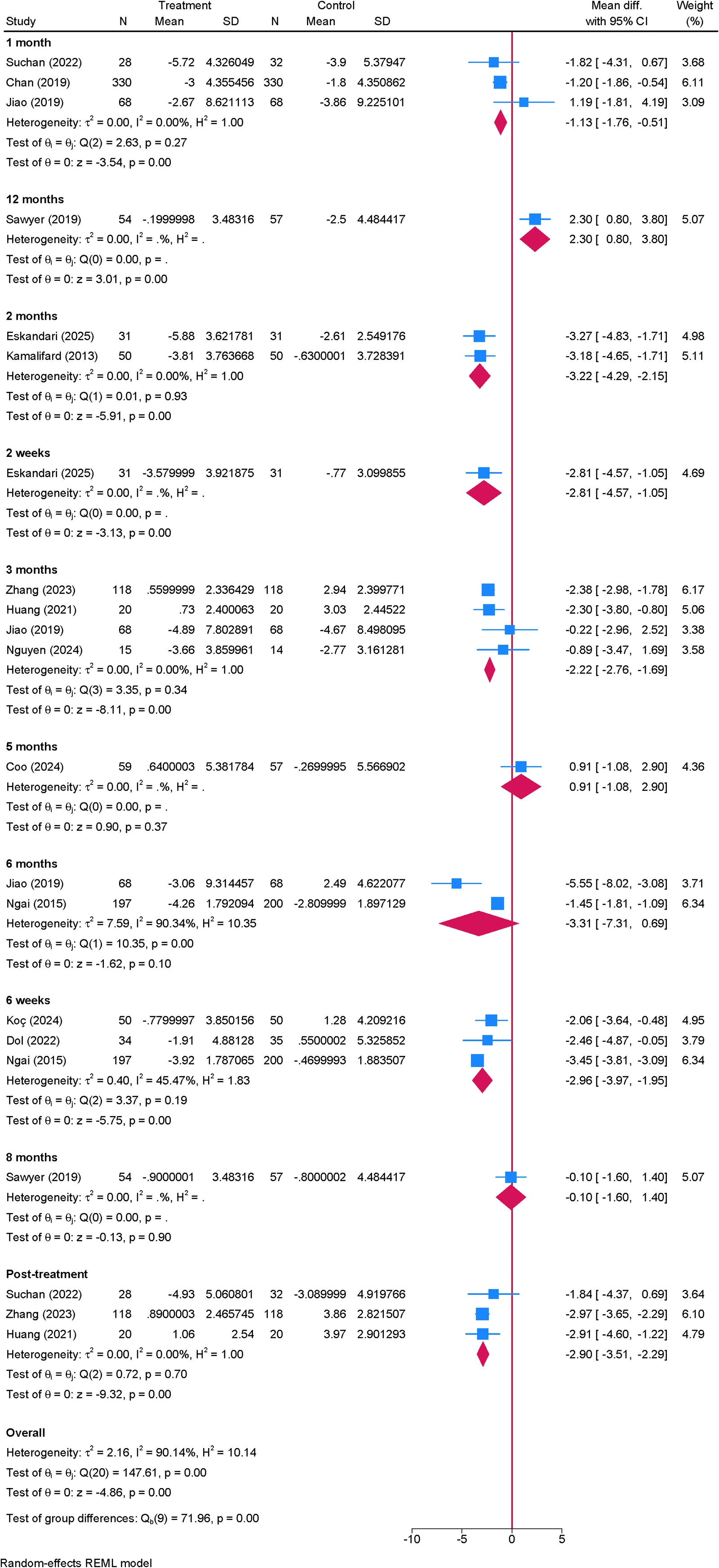
Forest plot of mean change in EPDS scores.

Subgroup analyses according to follow-up duration demonstrated variation in effect estimates across time points (Qb = 71.96, p < 0.001) (**Figure 4**).

At 1 month, telecounseling was associated with a statistically significant reduction in depressive symptoms (MD = −1.13, 95% CI −1.76 to −0.51; p < 0.001), with no observed heterogeneity (I² = 0%). Similarly, at 2 months, the pooled estimate favored telecounseling (MD = −3.22, 95% CI −4.29 to −2.15; p < 0.001) without heterogeneity.

At 3 months, telecounseling was associated with a reduction in EPDS scores (MD = −2.22, 95% CI −2.76 to −1.69; p < 0.001), with no evidence of heterogeneity (I² = 0%). At 6 weeks, the pooled estimate also favored telecounseling (MD = −2.96, 95% CI −3.97 to −1.95; p < 0.001), with moderate heterogeneity (I² = 45.47%).

At 2 weeks, a statistically significant reduction was observed (MD = −2.81, 95% CI −4.57 to −1.05; p < 0.001), although this estimate was based on a single study. At 6 months, the pooled estimate did not reach statistical significance (MD = −3.31, 95% CI −7.31 to 0.69; p = 0.10), with substantial heterogeneity (I² = 90.34%). No statistically significant differences were observed at 5 months (p = 0.37) or 8 months (p = 0.90). At 12 months, a statistically significant difference was observed favoring the control group (MD = 2.30, 95% CI 0.80 to 3.80; p = 0.003), based on a single study.

#### 3.4.2 Heterogeneity and small-study effects

The Galbraith plot demonstrated dispersion of several studies beyond the confidence limits, consistent with the high I² value observed in the pooled analysis and suggesting that a subset of studies contributed disproportionately to between-study variability (**Supplementary Figure 3**).

Visual inspection of the funnel plot showed some asymmetry in the distribution of effect sizes relative to their standard errors (**Supplementary Figure 4**); however, no significant risk of bias was observed (p = 0.3662).

#### 3.4.3 Meta-regression analysis

Follow-up duration was significantly associated with effect size, with increasing duration associated with more positive (less negative) change-score effect estimates (coefficient = 0.0807 per week; 95% CI 0.0357 to 0.1258; p < 0.001). Studies conducted in Australia demonstrated significantly different effect estimates compared with studies conducted in China (coefficient = 3.4542; 95% CI 1.5962 to 5.3122; p < 0.001). No statistically significant associations were observed for maternal age (p = 0.086), proportion of vaginal delivery (p = 0.609), or risk of bias classification (p = 0.203). Differences between other countries and China were not statistically significant (**Table 3**).

**Table 3:** Meta-regression analysis of the determinants of mean change in EPDS score in telecounseling vs. control.

Characteristic	Coefficient	P	2.5% CI	97.5% CI
Age (per year)	0.2133	0.0860	-0.0304	0.4570
Vaginal delivery (per % increase)	-0.0274	0.6090	-0.1323	0.0776
Country (Reference: China)				
Turkey	0.2942	0.8220	-2.2661	2.8546
Canada	0.3071	0.7570	-1.6419	2.2560
Singapore	0.5244	0.6170	-1.5309	2.5798
Iran	-0.7435	0.3660	-2.3552	0.8681
Australia	3.4542	p < 0.001	1.5962	5.3122
Follow-up (per week)	0.0807	p < 0.001	0.0357	0.1258
Quality (Some concerns vs. Low risk)	-0.9279	0.2030	-2.3567	0.5009

P, P-value; CI, confidence interval; EPDS, Edinburgh Postnatal Depression Scale.

These findings suggest that follow-up duration and study location may partly explain heterogeneity in change-score estimates, whereas demographic characteristics and risk of bias were not significantly associated with effect magnitude.

## 4 Discussion

### 4.1 Principal findings

This systematic review and meta-analysis evaluated the effect of telecounseling interventions on depressive symptoms, measured by the EPDS, among primiparous women during the postpartum period. The pooled analyses demonstrated that telecounseling was associated with a reduction in depressive symptom scores compared with usual care, although the magnitude of effect varied across studies and substantial between-study heterogeneity was observed. Meta-regression analyses indicated that longer follow-up was associated with less negative effect estimates, and this finding should be interpreted cautiously. In contrast, maternal age, proportion of vaginal delivery, and risk of bias classification were not significantly associated with treatment effects. These findings support the potential role of remotely delivered psychological interventions in reducing postpartum depressive symptoms among first-time mothers while highlighting variability in treatment effects across settings and intervention characteristics.

### 4.2 Comparison with previous evidence

The present findings are consistent with prior systematic reviews and meta-analyses evaluating telemedicine and digital psychological interventions for postpartum depression. Nair et al. (6) reported that telemedicine interventions were associated with improvements in maternal depressive symptoms in most included trials, although methodological limitations such as attrition and lack of blinding were common. Similarly, Liu et al. (7) demonstrated that telemedicine interventions significantly reduced postpartum depression and anxiety across randomized trials, and Zhao et al. (5) reported a pooled reduction in EPDS scores favoring telehealth interventions. The magnitude and direction of effects observed in the present analysis fall within the range reported in these previous syntheses, suggesting consistency across different populations and intervention models.

More specifically, Pan et al. (9) evaluated online cognitive behavioral therapy and reported significant improvements in postpartum depression, particularly when interventions were delivered over longer durations and included professional guidance. The association between longer follow-up duration and less negative effect estimates observed in the present meta-regression should be interpreted cautiously. Likewise, a network meta-analysis by Haq et al. (10) ranked therapist-assisted internet-based cognitive behavioral therapy as the most effective non-pharmacological intervention for postpartum depression, further supporting the potential importance of structured psychological interventions delivered remotely. While the present review did not directly compare telecounseling modalities, the observed variability in effect estimates across studies may partly reflect differences in therapeutic content, intensity, and provider involvement.

Hanach et al. (8), who focused exclusively on telemedicine interventions delivered during the postnatal period, also reported a modest but statistically significant reduction in depressive symptoms. However, their analysis included mixed parity populations, whereas the present study focused specifically on primiparous women, who may experience distinct psychosocial challenges during the transition to motherhood. Mueller et al. (13) similarly reported favorable mental health outcomes associated with telehealth interventions for perinatal care in a scoping review conducted during the COVID-19 pandemic, although their analysis included diverse clinical outcomes and did not provide pooled estimates.

Broader literature on postpartum support interventions provides additional context for these findings. Aydın et al. (12) described beneficial effects of structured postpartum support, including tele-counseling and home-based interventions, on maternal psychological outcomes, although the evidence was largely narrative and not restricted to randomized trials. Alum et al. (4) highlighted psychotherapy, particularly cognitive behavioral therapy, as an effective treatment for perinatal depression while emphasizing persistent barriers to access, including stigma, limited availability of trained providers, and logistical challenges. Technology-based interventions have been proposed as a potential strategy to address these barriers. In addition, Lara-Cinisomo et al. (11) emphasized that technology-based mental health interventions may improve engagement among underserved populations, although subgroup analyses by ethnicity or socioeconomic status were not consistently reported in the trials included in the present review.

A direct comparison with previous syntheses further clarifies the contribution of the present review (**Table 4**). To our knowledge, earlier systematic reviews of telehealth or telecounseling interventions for postpartum depression have evaluated heterogeneous populations of mixed parity, whereas the present review is the first to restrict eligibility to primiparous women and to use the EPDS as a standardized continuous outcome. The direction and magnitude of effect observed here are broadly consistent with these earlier reviews. Zhao et al. (5) reported a pooled mean difference of approximately −3 EPDS points favoring telehealth, Hanach et al. (8) reported a mean difference of −1.81 points among postnatal women without prior mental disorders, and Nair et al. (6) and Liu et al. (7) likewise found that most or all included trials favored telemedicine. Several of these reviews searched a larger number of databases, including Embase, the Cochrane Library, CINAHL, PsycINFO, and Chinese-language databases, which is acknowledged as a relative limitation of the present search; however, the present review adds value through its parity-specific focus, its use of both post-intervention and change-score analyses, and its exploration of heterogeneity through meta-regression.

**Table 4:** Comparison of the present review with previous systematic reviews and meta-analyses of telehealth or telecounseling interventions for postpartum depression.

Review (first author, year)	Population (parity)	Databases searched (n)	RCTs (n)	Intervention modalities	Main finding on depressive symptoms
Present review (2026)	Primiparous (first-time) mothers only	PubMed, Scopus, Web of Science (3)	15	Telephone, mobile application, SMS, internet-based CBT/support	Significant reduction in EPDS scores vs control (z = −6.26, p < 0.001); effect varied by follow-up duration; high heterogeneity (I² = 96.8%)
Nair et al., 2018 (6)	Maternal depression, mixed parity (pregnancy to 12 months postpartum)	Cochrane, PubMed/MEDLINE, PsycINFO, Embase (4)	10	CBT, behavioral activation, psychoeducation (telephone/web)	8 of 10 trials showed significant post-intervention improvement in depression scores
Zhao et al., 2021 (5)	Women with PPD, mixed parity	PubMed, Cochrane, CINAHL, PsycINFO, CNKI, Wanfang (6)	NR	Telephone, web-based, mobile application	Significant EPDS reduction favoring telehealth (MD ≈ −2.99)
Hanach et al., 2021 (8)	Postnatal women without prior mental disorder, mixed parity	PubMed, Web of Science, Cochrane, ProQuest (4)	10 (7 pooled)	Web-based, telephone	Significant improvement (MD = −1.81, 95% CI −2.68 to −0.93)
Liu et al., 2022 (7)	Women with PPD, mixed parity	Cochrane, PubMed, Embase, Web of Science, CINAHL, PsycINFO (6)	20	Telephone, web-based, mobile application	Telemedicine significantly reduced postpartum depression and anxiety
Pan et al., 2025 (9)	Women with PPD, mixed parity	13 databases plus 2 trial registries	NR	Online cognitive behavioral therapy	Significant improvement; greater with longer duration and professional guidance

CBT, cognitive behavioral therapy; CI, confidence interval; EPDS, Edinburgh Postnatal Depression Scale; MD, mean difference; NR, not reported in the available source; PPD, postpartum depression; RCT, randomized controlled trial; SMS, short message service.

The meta-regression also indicated that trials conducted in Australia were associated with significantly larger reductions in EPDS scores than trials conducted in China, the reference category. A plausible interpretation is that intervention effects may be larger in higher-income, higher-education settings, where greater digital literacy, more reliable internet access, and better-developed telehealth infrastructure may support higher engagement, adherence, and intervention fidelity. Participants with higher educational attainment may also engage more readily with self-directed, internet-based content, and health systems in these settings may integrate telecounseling more closely with existing perinatal care pathways. These observations are nonetheless consistent with the broader literature, in which structured, professionally guided interventions delivered in well-resourced settings tend to produce more favorable outcomes. However, this country-level association should be interpreted with considerable caution. Each country was represented by only one or a small number of trials, so the comparison is ecological and is confounded with intervention modality, intervention intensity, follow-up duration, and baseline depression severity rather than reflecting a causal effect of national income or education. The apparent advantage in higher-income settings should therefore be regarded as hypothesis-generating, and dedicated trials in lower-resource settings are needed before any conclusion about the moderating role of socioeconomic context can be drawn.

### 4.3 Interpretation of heterogeneity

Substantial heterogeneity was observed across studies, which may reflect variability in intervention characteristics, participant populations, and study design. Telecounseling interventions differed considerably with respect to therapeutic approach, including cognitive behavioral therapy, behavioral activation, psychoeducation, and peer support, as well as differences in duration, intensity, and level of professional involvement. Previous evidence suggests that structured interventions with professional guidance may yield greater improvements in depressive symptoms, which may partly explain variability in treatment effects across studies (35). Differences in healthcare systems, cultural context, and baseline depression severity may also contribute to heterogeneity. Although meta-regression indicated an association between country of study and effect size, such ecological comparisons should be interpreted cautiously because they may reflect unmeasured contextual factors rather than true differences in intervention effectiveness. A formal subgroup analysis by intervention modality (e.g., telephone counseling, mobile application, text message, or internet-based cognitive behavioral therapy) was considered but not performed, for two main reasons. First, many trials delivered multicomponent or hybrid interventions that combined more than one modality (for example, an application incorporating messaging and guided counseling), so trials could not be assigned unambiguously to mutually exclusive modality categories. Second, once stratified by modality and follow-up time point, the number of trials in each category was small, which would have yielded unstable and potentially misleading pooled estimates. The wide publication span of the included trials (2013 to 2025) further reflects the evolution of available telecommunication technologies over this period and was retained because the underlying construct of remotely delivered, interactive psychological support has remained consistent. Rather than a categorical subgroup analysis, intervention-related variability was examined through random-effects modelling and meta-regression, and the inability to disaggregate effects by specific modality is acknowledged as a limitation. Future trials and reviews with larger numbers of studies per modality are needed to clarify which delivery formats are most effective.

### 4.4 Clinical implications

The findings of this review suggest that telecounseling may reduce depressive symptom severity among primiparous postpartum women when used in addition to routine care. Given the well-documented barriers to accessing in-person psychological services during the postpartum period, including time constraints, childcare responsibilities, and stigma, telecounseling may represent a feasible adjunct to existing postpartum mental health services. However, the observed effects should be interpreted cautiously, and telecounseling should not be considered a substitute for comprehensive psychiatric evaluation or treatment in women with severe depressive symptoms or complex psychiatric comorbidities. Furthermore, because the present analysis focused on continuous EPDS scores rather than diagnostic thresholds, the impact of telecounseling on the incidence or remission of clinically defined postpartum depression cannot be determined.

### 4.5 Strengths and limitations

This study has several methodological strengths, including the exclusive inclusion of randomized controlled trials, the focus on primiparous women, the use of both post-intervention and change-score analyses, and the exploration of heterogeneity through meta-regression. The analysis also incorporated multiple approaches to assess small-study effects and potential publication bias.

Nevertheless, several limitations should be considered. First, substantial heterogeneity was observed across studies, reflecting variability in intervention mechanisms, delivery platforms, and therapeutic intensity. Second, most included trials were single-blinded due to the nature of behavioral interventions, which may introduce performance bias. Third, depression outcomes were analyzed as continuous EPDS scores without examination of clinically defined diagnostic thresholds, limiting interpretation of effects on disease burden or remission rates. Fourth, intervention mechanisms were not stratified by therapeutic modality, and differential effectiveness across intervention types could not be evaluated. Fifth, most studies were conducted in middle- or high-income settings, which may limit generalizability to low-resource populations. Sixth, attrition rates varied across studies, and differential loss to follow-up may influence treatment estimates. Seventh, baseline depression severity and other clinical characteristics were inconsistently reported, precluding subgroup analyses. Eighth, although three complementary multidisciplinary databases were searched and supplemented by manual reference-list screening, Embase and the Cochrane Library were not searched, and the restriction to English-language publications may have introduced a degree of selection bias. Finally, although statistical methods were used to assess publication bias, these approaches cannot definitively exclude small-study effects.

### 4.6 Future research

Future research should prioritize high-quality randomized trials that compare specific telecounseling modalities, evaluate long-term outcomes, and report standardized measures of clinical remission based on validated EPDS cutoff thresholds. Greater consistency in reporting intervention characteristics, participant demographics, and baseline severity is also needed. In addition, studies should examine differential effectiveness across socioeconomic and cultural contexts and assess the sustainability of treatment effects beyond the early postpartum period.

## 5 Conclusion

Telecounseling interventions were associated with reductions in depressive symptom scores among primiparous postpartum women compared with routine care, although the magnitude of effect varied and substantial heterogeneity was present. The findings are consistent with prior evidence supporting telehealth-based psychological interventions for postpartum depression, but methodological variability and differences in intervention mechanisms limit definitive conclusions. Telecounseling may represent a potentially accessible adjunct to postpartum mental health care, and further rigorously designed trials are required to clarify its role in clinical practice.

## Data Availability

The original contributions presented in the study are included in the article/**Supplementary Material**. Further inquiries can be directed to the corresponding author.

## Supplementary material

Supplementary Figure 1 Funnel plot of post-intervention mean difference in EPDS scores.

Supplementary Figure 2 Galbraith plot of post-intervention mean difference in EPDS scores.

Supplementary Figure 3 Galbraith plot of mean change in EPDS scores.

Supplementary Figure 4 Funnel plot of mean change in EPDS scores.
