# Supplementary material for: Effect of telecounseling on depressive symptoms in primiparous women during the postpartum period: a systematic review and meta-analysis of randomized controlled trials
Source: Front Psychiatry. 2026 Aug 28;17:1863575. doi: 10.3389/fpsyt.2026.1863575 (PMC13561997; doi:10.3389/fpsyt.2026.1863575)
Supplement: Supplementary file 2 [file Image2.pdf]

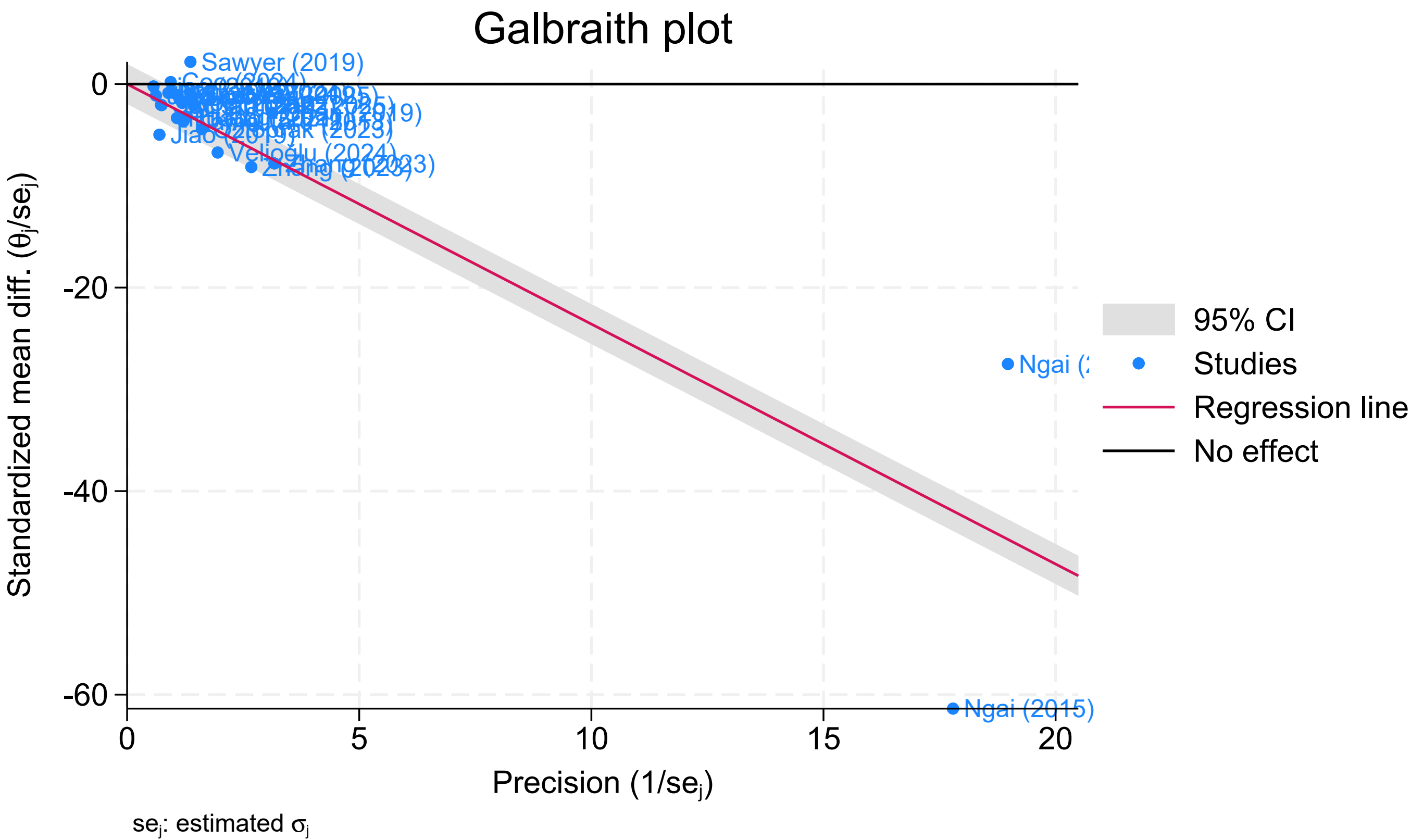

Galbraith plot
Sawyer (2019)
Jiao (2019)
Velioglu (2024)
Ngai (2015)
0
-20
-40
-60
0
5
10
15
20
Standardized mean diff. (θj/sej)
Precision (1/sej)
95% CI
Studies
Regression line
No effect
sej: estimated σj
