# Supplementary material for: Effect of telecounseling on depressive symptoms in primiparous women during the postpartum period: a systematic review and meta-analysis of randomized controlled trials
Source: Front Psychiatry. 2026 Aug 28;17:1863575. doi: 10.3389/fpsyt.2026.1863575 (PMC13561997; doi:10.3389/fpsyt.2026.1863575)
Supplement: Supplementary file 4 [file Image4.pdf]

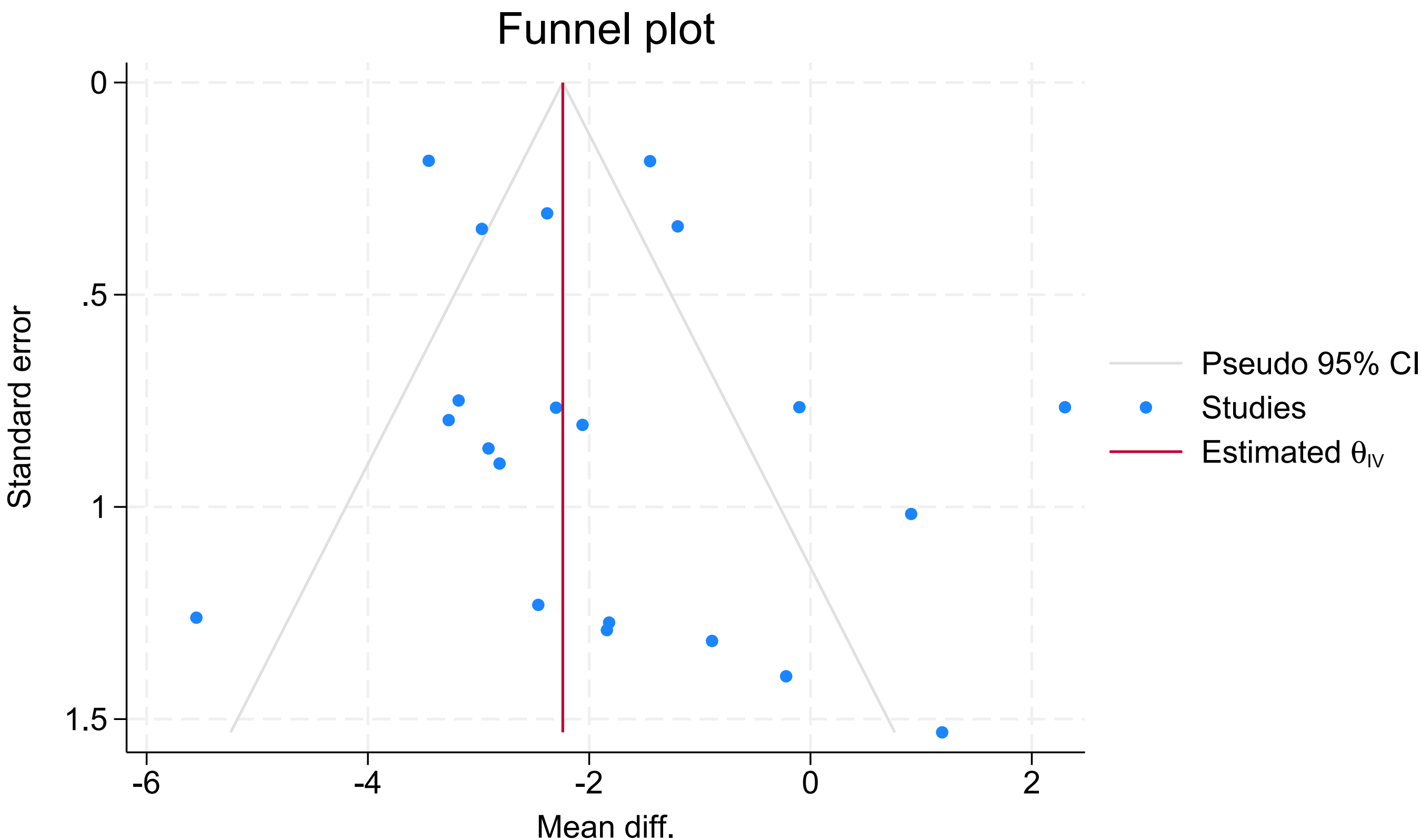
Funnel plot
Standard error
0
.5
1
1.5
-6
-4
-2
0
2
Mean diff.
Pseudo 95% CI
Studies
Estimated θ_{IV}
