# Supplementary material for: Effect of telecounseling on depressive symptoms in primiparous women during the postpartum period: a systematic review and meta-analysis of randomized controlled trials
Source: Front Psychiatry. 2026 Aug 28;17:1863575. doi: 10.3389/fpsyt.2026.1863575 (PMC13561997; doi:10.3389/fpsyt.2026.1863575)
Supplement: Supplementary file 5 [file Table1.docx]

**Table S1.** The search query employed in the database search

| No. | Search Query | Results |
| --- | --- | --- |
| PubMed | | |
| #1 | Telecounseling[tiab] OR “tele-counseling”[tiab] OR telehealth[tiab] OR tele-health[tiab] OR “mobile application”[tiab] OR telecare[tiab] OR tele-care[tiab] OR ehealth[tiab] OR e-health[tiab] OR telemedicine[tiab] OR tele-medicine[tiab] OR telecommunication[tiab] OR tele-communication[tiab] OR online[tiab] OR internet[tiab] OR telephone[tiab] OR mobile[tiab] OR web-based[tiab] OR "Telemedicine"[Mesh] | 725448 |
| #2 | Primipar*[tiab] OR “first-time”[tiab] OR new[tiab] OR “first time”[tiab] | 3834383 |
| #3 | Postpartum[tiab] OR postnatal[tiab] OR post-partum[tiab] OR post-natal[tiab] OR maternal[tiab] | 548415 |
| #4 | Depression[tiab] OR "Depression"[Mesh] OR EPDS | 551493 |
| #5 | Random*[tiab] OR "Randomized Controlled Trial" [Publication Type] | 1865727 |
| #6 | #1 AND #2 AND #3 AND #4 AND #5 | 105 |
| Scopus | | |
| #1 | TITLE-ABS-KEY(Telecounseling) OR TITLE-ABS-KEY(“tele-counseling”) OR TITLE-ABS-KEY(telehealth) OR TITLE-ABS-KEY(tele-health) OR TITLE-ABS-KEY(“mobile application”) OR TITLE-ABS-KEY(telecare) OR TITLE-ABS-KEY(tele-care) OR TITLE-ABS-KEY(ehealth) OR TITLE-ABS-KEY(e-health) OR TITLE-ABS-KEY(telemedicine) OR TITLE-ABS-KEY(tele-medicine) OR TITLE-ABS-KEY(telecommunication) OR TITLE-ABS-KEY(tele-communication) OR TITLE-ABS-KEY(online) OR TITLE-ABS-KEY(internet) OR TITLE-ABS-KEY(telephone) OR TITLE-ABS-KEY(mobile) OR TITLE-ABS-KEY(web-based) | 3618908 |
| #2 | TITLE-ABS-KEY(Primipar*) OR TITLE-ABS-KEY(“first-time”) OR TITLE-ABS-KEY(new) OR TITLE-ABS-KEY(“first time”) | 13042126 |
| #3 | TITLE-ABS-KEY(Postpartum) OR TITLE-ABS-KEY(postnatal) OR TITLE-ABS-KEY(post-partum) OR TITLE-ABS-KEY(post-natal) OR TITLE-ABS-KEY(maternal) | 796996 |
| #4 | TITLE-ABS-KEY(Depression) | 1056714 |
| #5 | TITLE-ABS-KEY(Random*) | 3794115 |
| #6 | #1 AND #2 AND #3 AND #4 AND #5 | 140 |
| Web of Science | | |
| #1 | AB=Telecounseling OR AB=“tele-counseling” OR AB=telehealth OR AB=tele-health OR AB=“mobile application” OR AB=telecare OR AB=tele-care OR AB=ehealth OR AB=e-health OR AB=telemedicine OR AB=tele-medicine OR AB=telecommunication OR AB=tele-communication OR AB=online OR AB=internet OR AB=telephone OR AB=mobile OR AB=web-based | 1846996 |
| #2 | AB=Primipar* OR AB=“first-time” OR AB=new OR AB=“first time” | 8453967 |
| #3 | AB=Postpartum OR AB=postnatal OR AB=post-partum OR AB=post-natal OR AB=maternal | 500006 |
| #4 | AB=Depression | 529999 |
| #5 | AB=Random* | 2528236 |
| #6 | #1 AND #2 AND #3 AND #4 AND #5 | 79 |
